# Supplementary figures and images for: Breast Digital Tomosynthesis versus Contrast-Enhanced Mammography: Comparison of Diagnostic Application and Radiation Dose in a Screening Setting
Source: Cancers (Basel). 2023 Apr 22;15(9):2413. doi: 10.3390/cancers15092413 (PMC10177523; doi:10.3390/cancers15092413)

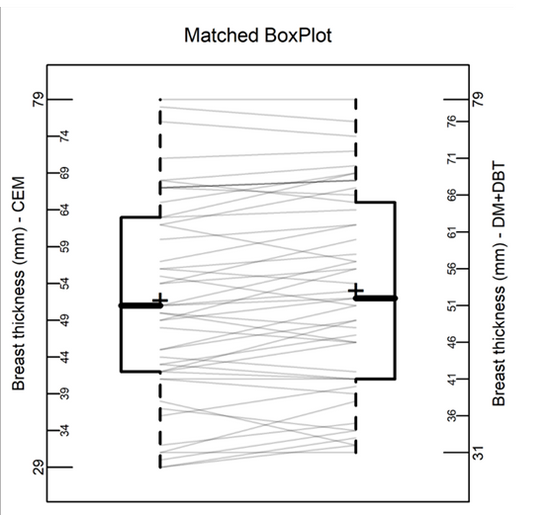

Supplement: Supplementary file 1 [file cancers-15-02413-s001.zip › Figure S1.tif]
